# Geochemistry and Evolutionary Characteristics of Rare Earth Elements in Ordovician Carbonate-Evaporite Rocks of the Central-Eastern Ordos Basin, Central China

**DOI:** 10.1021/acsomega.5c03353

**Published:** 2025-12-25

**Authors:** Hongping Bao, Baohong Shi, Zhanrong Ma, Liubin Wei, Ting Yan, Wei Yan, Yan Zhang

**Affiliations:** † Exploration and Development Institute, 631241 Changqing Oil Field Branch, Petrochina Company Limited, Xi’an 710018, China; ‡ National Engineering Laboratory for Exploration and Development of Low Permeability Oil & Gas Fields, Xi’an 710018, China; § School of Earth Sciences and Engineering, Xi’an Shiyou University, Xi’an 710065, China

## Abstract

This study aims to reveal the geochemical behavior characteristics of rare earth elements (REEs) in the process of seawater evaporation and concentration by analyzing the REEs and their variation characteristics in different stages of rocks from the rock system of carbonate-gypsum-salt (C-G-S system) of the Ordovician in the central-eastern Ordos Basin, China. The results indicate: despite the low overall content of REEs in the endogenous deposition process of the C-G-S system in this area, a relatively consistent REEs distribution pattern is still exhibited in different types of rocks formed in different stages of seawater evaporation and concentration, showing a relative enrichment of light rare earth elements (LREEs) and obvious negative europium (Eu) anomalies; and its REEs content is not directly related to the content of continental source detrital minerals. All this means that REEs do not enter the crystal structure of the main rock-forming minerals mainly in the form of isomorphous substitution during the mineral crystallization process but are more likely to aggregate in the form of extremely fine particles, exist in the lattice defects and mineral surfaces of the main rock-forming minerals, or be enclosed by subsequent growth of mineral crystals. Also, during the dolomitization transformation from limestone to dolomite, REEs undergo relatively significant outward migration.

## Graphical abstract

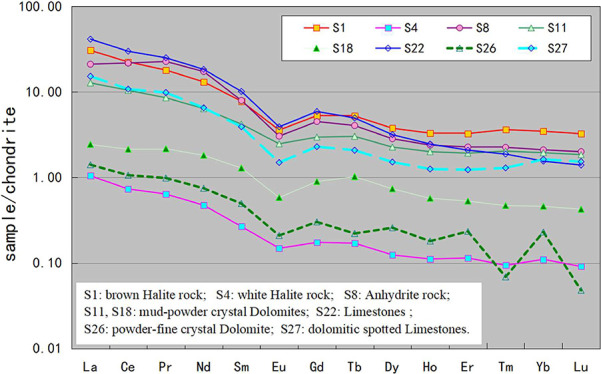

## 1 Introduction

According to the study of modern oceanography, Except for oxygen and hydrogen elements that constitute water, other elements in seawater mainly exist in ionic, the main ions with high content are chloride (Cl^–^), sodium (Na^+^), sulfate (SO_4_^2–^), magnesium (Mg^2+^), calcium (Ca^2+^), potassium (K^+^), and bicarbonate (HCO_3_^–^); in addition, there are small amounts of Fe, Mn, Si, Al, P, Ti, and trace amounts of Sr, Ba, Y, U, and rare earth elements, etc. Among them, Chloride (Cl^–^) and Sodium (Na^+^) account for 85% of the dissolved ions.^1–3^ However, limited by the evaporation concentration degree of seawater and the solubility of minerals in water and other factors, the sequence of crystallization precipitation of salt minerals in the process of seawater evaporation concentration is generally carbonate-sulfate-chloride.^4,5^ That is, carbonate minerals, which are less in content in seawater, usually crystallize and precipitate first, followed by sulfate minerals, whereas minerals such as chloride (Cl^–^) and sodium (Na^+^), which are very high in content, crystallize and precipitate in the very late stage.

The geochemica study on major and trace elements in sedimentary rocks often uses the content of specific elements or their ratios to indicate some key factors of the ancient sedimentary environment, such as using the chemical index of weathering (CIW = Al_2_O_3_/(Al_2_O_3_ + CaO* + Na_2_O) × 100) to indicate the weathering and paleoclimate characteristics;^6–8^ using the content of Sr or Sr/Ba ratio to indicate the paleosalinity of the depositional water body;^9–11^ using the content of P or P/Ti ratio to indicate paleo-bioproductivity;^12–15^ using redox-sensitive elements (V, Co, U, and Ni) to reconstruct ancient depositional environments;^16–18^ using U/Th ratio to indicate paleo-redox conditions;^15^ using the content of Fe, Mn elements and their valence state changes to indicate the redox characteristics of the depositional environment;^19–21^ using Y/Ni with Cr/V, Th/Sc, Th/Co, La/Co, La/Sc, or Al/Si versus Fe + Mn, etc. to characterize the composition of sediment provenance and tectonic setting.^22–25^ However, most of such studies have been concentrated on terrigenous clastic rocks, whereas endogenous sedimentary rocks have relatively few studies concerned with them.

For the geochemical behavior characteristics of constant elements and some trace elements such as Fe, Mn, Sr, Ba, Y, U, etc., in the process of endogenous chemical deposition, some research work has been done by predecessors.^26–35^ Subsequent studies on trace elements by modern researchers have also proposed some new insights, such as the proposition that in the surficial environment (mainly in aqueous solution), trace elements are mainly distributed in the solution in the form of ions and colloids, or adsorbed on fine particulate matter as inner-sphere complexes or outer-sphere complexes.^36–38^ The distribution coefficient of trace elements in the adsorption process is also affected by a variety of factors such as the composition of solid phase particulate matter, the composition of aqueous solution, and redox conditions.^39,40^ Other researchers have proposed that the content of divalent iron ions (Fe_carb_) in the carbonate lattice can be used to trace the intensity of the seafor flux, indicating the redox conditions of the seafloor, and high Fe_carb_ can be used as a marker of hypoxic environments.^41^

In addition, during the normal process of endogenous chemical deposition, there is usually a small amount of continental clastic material and volcanic clastic material added. The content of Al and Ti in carbonate rocks can often be used as an important parameter to identify the influence of clastic material.^42–44^ The abnormal content of elements such as U and Y can usually be used as more reliable information for the addition of volcanic debris material.^45–50^

Rare Earth Elements (REEs) form a distinctive group of elements characterized by their tendency to occur together in geological formations. Their stable trivalent ionic forms and similar physical and chemical properties facilitate their utilization in tracing sedimentary processes and crustal compositions. As indicators of terrigenous input, REEs, particularly lanthanide elements, pose significant importance in sedimentological studies, especially in understanding depositional environments and reconstructing paleogeographical conditions. In the context of marine environments, the behavior and distribution of REEs can illuminate sedimentary processes, contributing to our knowledge of ancient marine conditions and the origins of mineral deposits.

The use of trace elements (including rare earth elements) in sediments to trace the properties of sediment source areas, crustal composition, and evolution was first proposed by Taylor and Mclennan,^51^ and it quickly gained widespread application.^52–54^ Shortly after the Taylor model was proposed, many scholars^55–60^ challenged the Taylor model and made sharp criticisms of Taylor’s work. The most strongly questioned one is that the Taylor model ignores the influence of sedimentary environment on sediment REE, which was considered a fatal mistake of the Taylor model.^61,62^

The unique geochemical properties of REE make them particularly useful in studies of marine geochemistry. They are an extremely coherent group, so that their relative abundances can be used to deduce their sources in sedimentary deposits, and their subtle changes in element distribution characteristics can also have a certain indicative effect on marine sedimentary environments. As the study of rare earth elements, trace elements and organic matter content of the shales from Wufeng-Longmaxi Formations (formed in Late Ordovician-Early Silurian marine environment) in the Yangtze Platform (Southern China) by Xiao et al. has shown, the parent rock of these deposits is characterized by granite source, the climate was warm and humid during the forming of Wufeng-Longmaxi, and the organic matter was enriched under hypoxia-anoxia marine environment.^63–65^

Despite substantial advances in REE research, particularly in clastic sedimentary systems, comprehensive studies of REEs in endogenous carbonate and evaporative systems remain limited. Previous studies^66–69^ have examined REE behavior in various settings, yet little systematic research has investigated their geochemical characteristics in the context of carbonate-gypsum-salt rock systems. Notably, the influence of exogenous materials is diminished in endogenous sedimentary environments, thereby enhancing the importance of REE characteristics in interpreting sedimentary processes. This work aims to address this gap by providing a detailed analysis of REE behavior in the Ordovician carbonate-evaporite system of the Ordos Basin, highlighting the geochemical evolution and implications for sedimentary dynamics.

This article analyzed the changes in the content of rare earth elements in various mineral phases formed in different crystallizing stages due to the normal seawater evaporating to concentrated seawater in the Ordovician North China Sea from the perspective of geochemistry and studied the morphological characteristics of the distribution curve of REE to investigate the geochemical behaviors of rare earth elements during the deposition and crystallization evolution of C-G-S rock system. And it can provide new research ideas and clues for exploring the sedimentary petrology of evaporite minerals.

## 2 Geological Settings

### 2.1 Paleogeographic Patterns of the Ordovician in the Ordos Basin

The Ordos Basin was located in central China (Figure 1). It is a multicyclic superposition basin that developed on the crystal base of the Archaeology-Paleoproterozoic.^70–76^ During the Early Paleozoic, it was located on the southwestern margin of the North China Paleo-Continent Block.^77,78^

**Figure 1:**
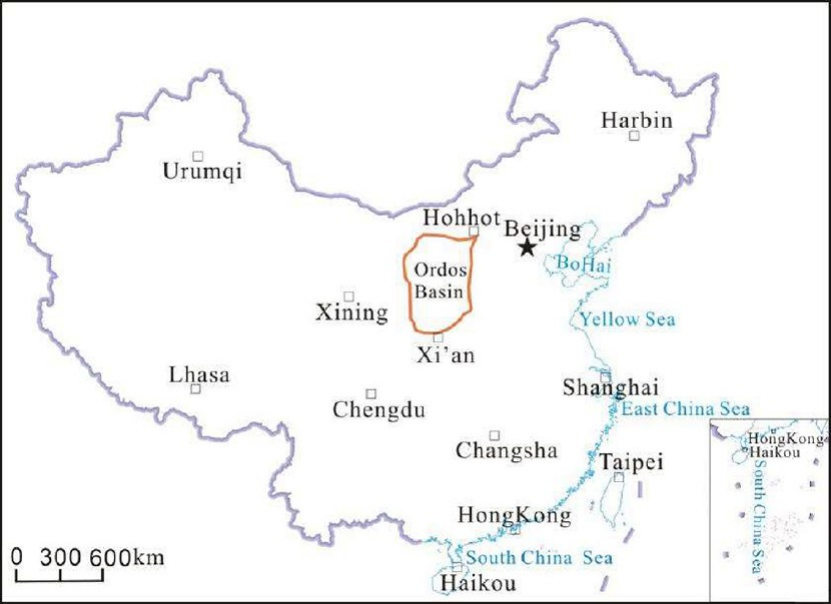
Location of the Ordos Basin in China’s map (the red contour line indicates the location of the Ordos Basin).

The Ordovician tectonic-sedimentary differentiation was relatively strong in the Ordos Block, forming a paleo-tectonic pattern of alternating distribution between uplift and depression.^79^ In the mideastern depression, there is the development of a hugely thick carbonate rock and evaporite symbiotic sedimentary system in the Majiagou Formation, with a cumulative stratigraphic thickness of 600–900 m. It is a set of cyclic strata of carbonate rock interbedded with gypsum salt rock (abbreviated as C-G-S rock system), whose distribution covers most of the mideastern Ordos Basin, with an area of more than 100,000 km^2^ (Figure 2). It has attracted widespread attention from researchers in sedimentology and oil and gas exploration, and salt resources exploration departments, because the natural gas resources and salt mineral resources contained therein are relatively abundant.^80–85^ However, on the whole, the sedimentary geochemistry of the Ordovician carbonate rock and gypsum-salt rock symbiotic system in this area is less researched.

In the early Paleozoic, the Ordos Basin began to subside as a whole and accepted marine sedimentation. There was a large-scale paleo-continent in the north of the basin, namely, the Ulanger paleo-continent (also called Yimeng paleo-continent), and an L-shaped, intermittently exposed paleo-uplift (Central paleo-uplift) had developed in the southwestern part of the basin. An underwater uplift (Lvliang paleo-uplift) was developed in the eastern margin of the basin. On the western and southern sides of the Central uplift were the open sea sedimentary areas of the Qilian Sea and Qinling Sea. And the east side of the Central uplift was the epicontinental marine sedimentary area of the North China Sea. There was a subsidence area of relative depression between the Central paleo-uplift and the Lvliang paleo-uplift, which is called the northern Shaanxi salt depression.

During the Ordovician depositional period, the northern Ulanger paleo-continent (Yimeng paleo-continent), Central paleo-uplift, Lvliang Paleo-uplift, and other structures played a decisive role in controlling the paleogeographic pattern of the Ordos region, and in turn controlled the sedimentary facies in different periods and regional lithology distribution pattern.^79,86^

First of all, the enclosure of the paleocontinent and paleo-uplift controlled the formation of the salt depression basin in northern Shaanxi. This was particularly prominent during the regressive sedimentation period. At this time, since the surrounding paleo-continent and paleo-uplift areas were mostly exposed to the surface, which played an important role in isolating the open sea, this sedimentary pattern resulted in the formation of regional lithology distribution pattern from halite depression to gypsum slope and gypsum-bearing dolomitic flat, which developed in sequence outward by taking the northern Shaanxi salt depression (Mizhi salt depression) as the center,^87^ see Figures 2 and 3 for more details.

**Figure 2:**
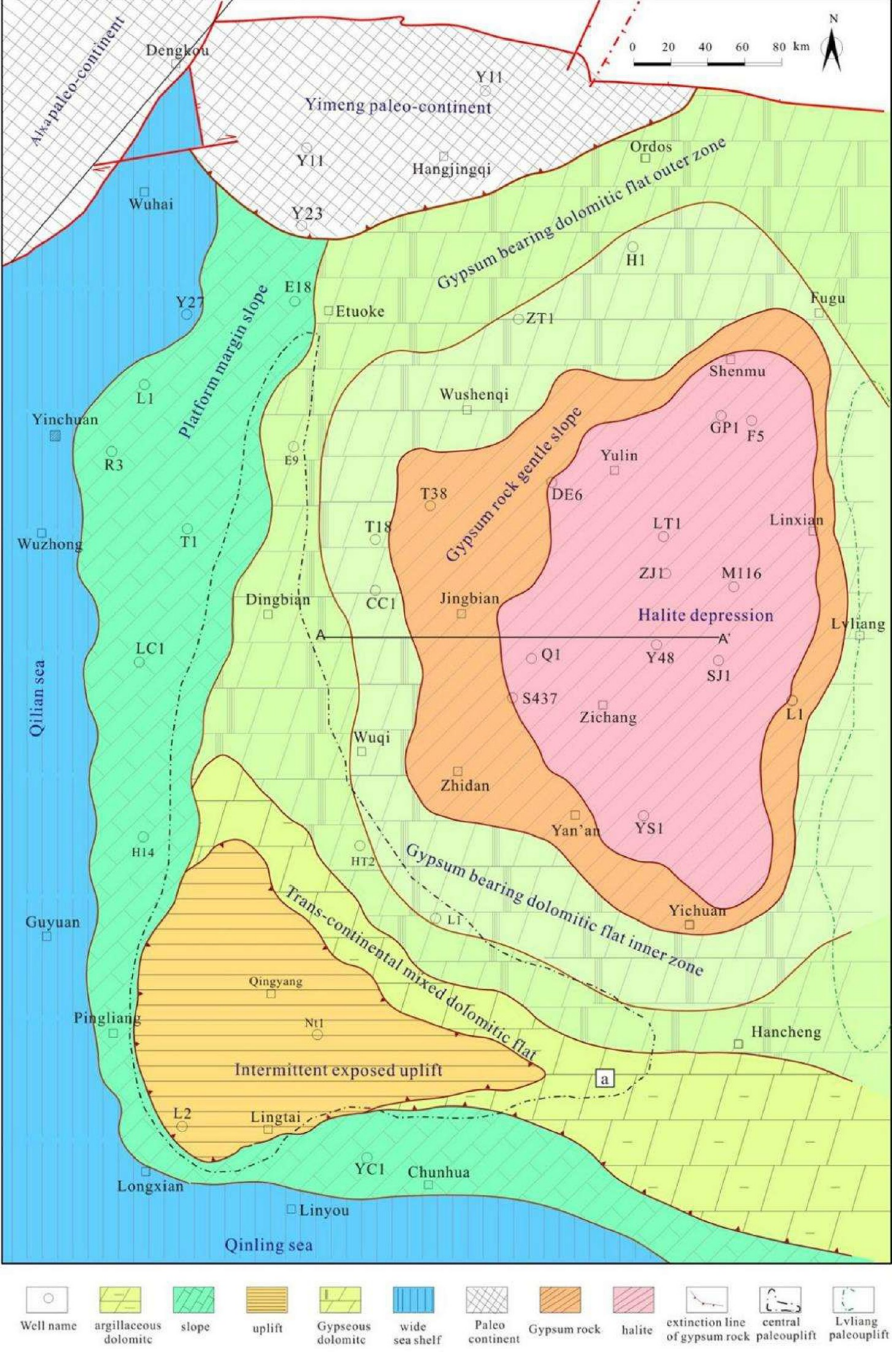
Lithofacies and paleogeographic map for Ma 5 chron in the Ordos Basin (the position of the section line of Figure 3 is shown by the line A–A′).

**Figure 3:**
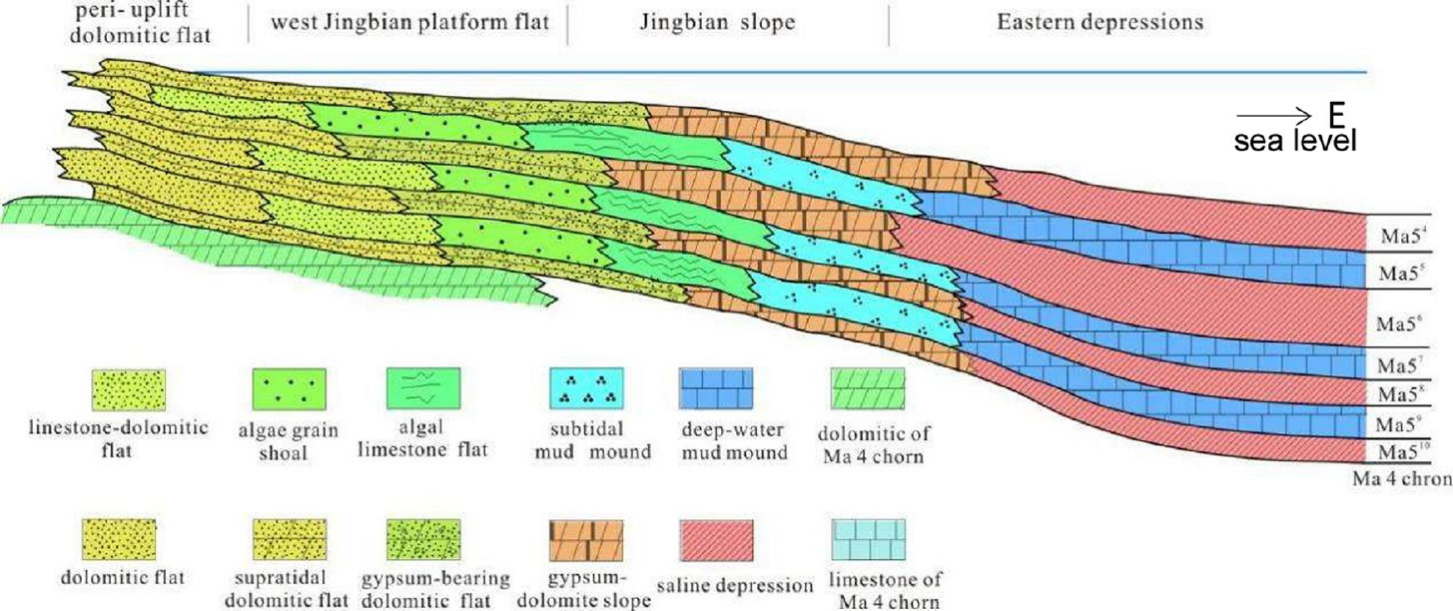
Sedimental model map of the early middle Ma 5 chron in central-eastern Ordos (the position of the section line see the line A–A′ in Figure 2).

Second, the Central paleo-uplift during the marine transgression period prominently controlled the regional lithological facies changes in the mideastern Ordos Basin. During the transgression period, the sea level rose sharply, and the role of the paleo-uplift barrier to the open sea was greatly reduced. As a result, most areas of the Ordos Basin (including the salt depression sedimentary area in the regression period) were mainly carbonate rock deposits. At this time, the Central paleo-uplift still played an important role in controlling the regional lithological facies changes in the mideastern areas of the basin. From the Central paleo-uplift to the east, shallow platform granular shoal facies, central lime-dolomite gentle slope facies, and east, darker lime mud depressions were developed in sequence. Sedimentation and lithology also showed the regional lithological facies change law of dolomite–dolomite intercalated with limestone–limestone (Figure 3)

### 2.2 Cyclostratigraphy of the Majiagou Formation of the Ordovician

At the end of the Cambrian, the Ordos area experienced a short period of uplift and denudation, which was called the Xinkai Movement in China. And after this depositional break, a new round of transgressive deposition began from the Early Ordovician. However, the range of initial transgression deposits in the Yeli-Liangjiashan period (O_1_y-O_1_l age) was relatively limited in the semiannular area in the western, southern, and eastern margins of Ordos, in which endogenous carbonate deposits of coastal neritic—open sea shelf facies were mainly developed.

During the Majiagou Formation period, under the influence of regional sea level rise, the extent of marine intrusion began to expand rapidly, forming extensive marine deposits that covered most of the Ordos Basin. The Central paleo-uplift located in the midwestern region of the Ordos Basin is an important “watershed” of the Ordovician deposition. There is a restricted sea platform facies deposition in the east of the Central paleo-uplift, but in the west of the Central paleo-uplift is a deep water slope-abyssal basin facies deposition of a normal wide sea.

The most prominent feature of restricted sea platform deposition in the central and eastern regions is the cyclonic sedimentary buildup of carbonate rocks and evaporative gypsum salt rocks. Upwardly, the Majiagou Formation in this area can be divided into 6 members, including Ma 1, Ma 2, Ma 3, Ma 4, Ma 5, and Ma 6. The Ma 1, Ma 3, and Ma 5 Members are mainly gypsum salt rock and evaporative tidal flat dolomite, which represent the sedimentary characteristics of the restricted sea evaporation environment during the regression period. However, the Ma 2, Ma 4, and Ma 6 Members are dominated by carbonate rocks, with a small amount of anhydrite intercalation, which represent the sedimentary characteristics of the open sea during the transgression period when the water bodies of the Qilian-Qinling sea and the North China sea were basically connected. Three major stratigraphical cycles are formed from Ma 1 to Ma 6 (Figures 4 and 5), which contain the subcycles in each member. Taking the Ma 5 Member as an example, among the ten submembers divided from top to bottom, the Ma 5^1–3^, Ma 5^5^, Ma 5^7^, and Ma 5^9^ are dominated by carbonate strata representing short-term marine transgression sedimentation. However, Ma 5^4^, Ma 5^6^, Ma 5^8^, and Ma 5^10^ are dominated by gypsum salt deposition during the regression period. All these reflected the frequent sea-level rise and fall cyclic features of the Majiagou Formation period.

**Figure 4:**
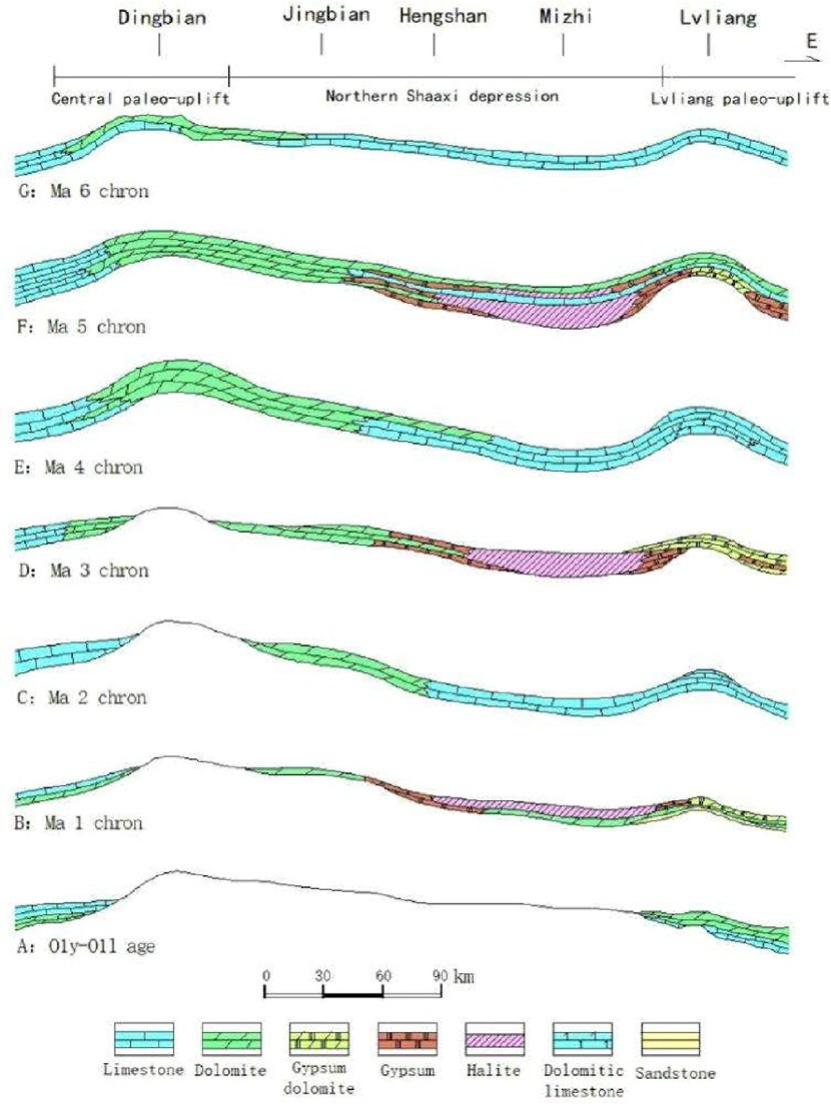
Sedimentary evolution of the Early Ordovician in the Ordos Basin.

**Figure 5:**
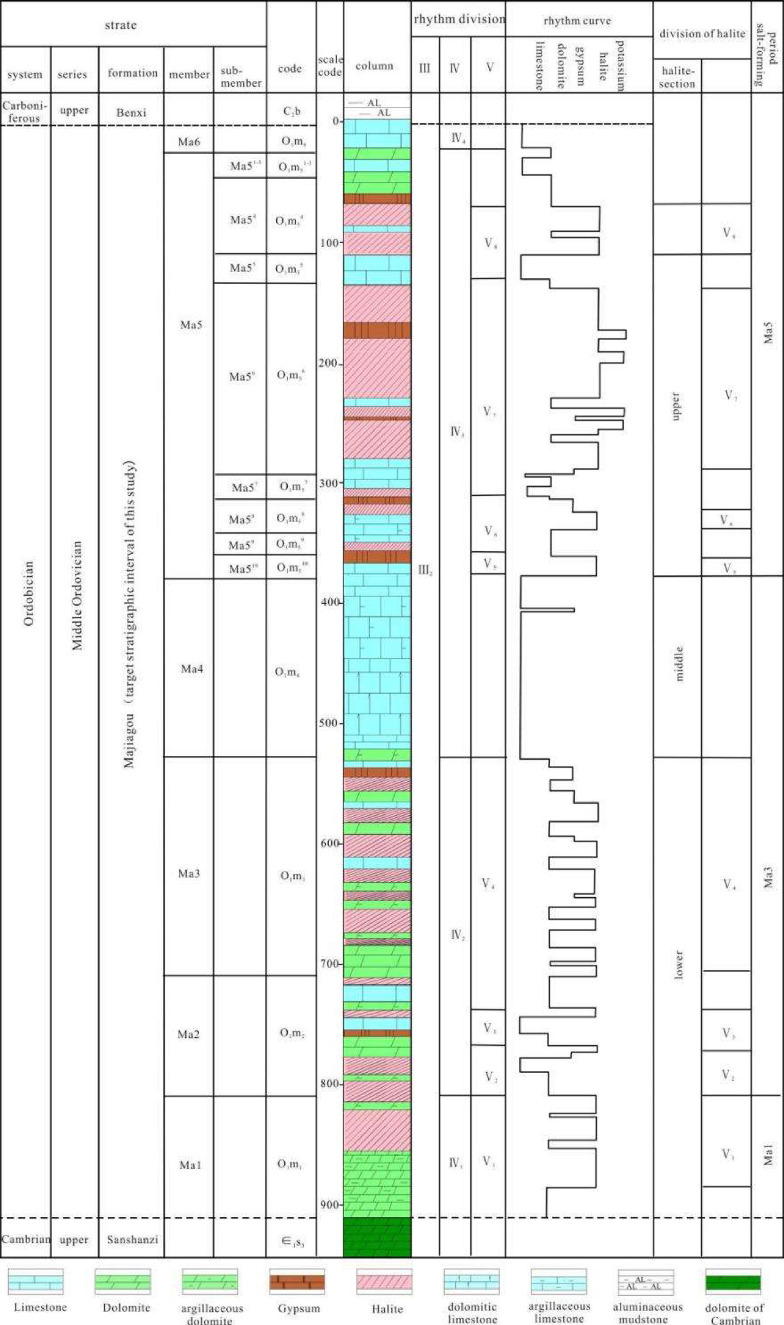
Columnar section of the Ordovician Majiagou Fm. evaporate-carbonate sedimentary cycle.

The target stratigraphic interval of this study is the Majiagou Formation of the Ordovician. In the main part of the Ordos Basin, the lower and upper series of the Ordovician are missing in this study area, and only the Majiagou Formation of the middle series of Ordovician is developed.

## 3 Rock, Mineral, and Geochemical Analysis

### 3.1 Categories of Analysis

The purpose of this study is to investigate the geochemical behaviors of rare earth elements in carbonate rock and the gypsum-salt rock system. For this purpose, it is necessary to understand its basic rock types, mineral composition, and fabric characteristics first. Therefore, the thin section observation on the rocks was carried out in this study to analyze the rock fabric and mineral composition, and scanning electron microscope (SEM) analysis on the rocks was also carried out in this study to analyze the microstructure characteristics of the rocks, with the help of a matching energy spectrometer to help identify the mineral composition.

Second, it is necessary to clarify the major chemical compositions of the rocks that the REEs host, so the contents of constant elements of each rock type were analyzed to clarify its main chemical compositions and mineral compositions (see Table 1). In order to provide some possible clues for the analysis of the changes in rare earth element content in different rock types, two sets of trace elements with special indicative significance were also selected to be analyzed. Sr and Ba elements are sensitive to changes in seawater salinity during evaporation and concentration processes; The other elements are U and Y, which are sensitive to external substances (especially volcanic ash, etc.).

**Table 1:** Analytical Table of Contents of Major and Trace Elements in the Major Rock Types in the Carbonate Rock-Gypsum Rock System^a^

	sample information			
sample no.	well no.	well depoth (m)	geologic horizon	lithology
S1	GP1	2440.57	Ma 5^6^	brown halite rock
S2	GP1	2469.78	Ma 5^6^	
S3	GP1	2476.58	Ma 5^6^	
S4	GP1	2442.00	Ma 5^6^	white, grayish halite rock
S5	GP1	2470.13	Ma 5^6^	
S6	GP1	2474.09	Ma 5^6^	
S7	GP1	2473.65	Ma 5^6^	
S8	SH 473	3746.95	Ma 5^6^	anhydrite rock
S9	SH 473	3744.83	Ma 5^6^	
S10	SH 473	3744.57	Ma 5^6^	
S11	GP1	2450.23	Ma 5^6^	mud-powder crystal dolomite
S12	GP1	2450.40	Ma 5^6^	
S13	M116	2424.44	Ma 5^5^	
S14	M116	2425.17	Ma 5^5^	
S15	M116	2425.88	Ma 5^5^	
S16	M116	2425.97	Ma 5^5^	
S17	M116	2424.65	Ma 5^5^	
S18	M116	2424.19	Ma 5^5^	
S19	M116	2423.71	Ma 5^5^	limestone
S20	M116	2423.59	Ma 5^5^	
S21	M116	2423.47	Ma 5^5^	
S22	M116	2423.09	Ma 5^5^	
S23	M116	2424.37	Ma 5^5^	
S24	M116	2423.22	Ma 5^5^	
S25	SH 473	4023.34	Ma 4	powder-fine crystal dolomite
S26	SH 473	4026.25		
S27	SH 473	4030.13	Ma 4	dolomitic spotted limestone
S28	SH 473	4039.17	Ma 4	

	major element content (%)										
sample no.	SiO_2_	Al_2_O_3_	MgO	Na_2_O	NaCl	K_2_O	P_2_O_5_	TiO_2_	CaO	TFe_2_O_3_	MnO
S1	10.49	3.33	3.86	/	45.21	0.68	0.04	0.13	10.84	0.61	0.01
S2	3.97	1.14	1.53	/	61.97	0.41	0.02	0.07	9.72	0.25	0.00
S3	8.56	2.64	2.78	/	69.08	0.67	0.04	0.10	4.41	0.63	0.00
S4	0.68	0.11	0.18	/	98.40	0.13	0.01	0.03	0.49	0.03	0.00
S5	1.04	0.26	0.32	/	94.60	0.13	0.01	0.04	1.25	0.06	0.00
S6	2.50	0.45	0.54	/	94.00	0.25	0.01	0.04	0.84	0.11	0.00
S7	0.64	0.09	0.07	/	98.49	0.07	0.01	0.03	0.64	0.03	0.00
S8	4.75	0.66	10.63	*	/	0.20	0.01	0.03	52.78	1.04	0.01
S9	9.89	2.49	7.07	*	/	1.07	0.03	0.09	52.02	0.76	0.01
S10	12.20	3.33	4.59	*	/	1.34	0.05	0.16	53.14	1.06	0.01
S11	3.97	0.79	13.77	/	25.75	0.25	0.02	0.06	18.72	0.35	0.00
S12	9.65	2.96	15.57	/	13.17	0.69	0.04	0.13	19.30	0.91	0.01
S13	14.13	4.34	15.72	0.22	/	2.11	0.07	0.26	23.67	1.54	0.02
S14	23.65	5.39	11.40	0.40	/	2.01	0.11	0.37	23.26	2.66	0.02
S15	14.88	4.89	15.79	0.42	/	1.65	0.05	0.30	22.93	1.17	0.01
S16	9.53	0.63	17.18	1.99	/	0.54	0.01	0.02	26.97	0.54	0.01
S17	1.33	0.27	19.37	2.39	/	0.22	0.01	<0.01	28.97	0.54	0.01
S18	1.24	0.32	19.91	0.20	/	0.17	0.00	0.01	32.32	0.77	0.01
S19	1.08	0.33	0.90	*	/	0.11	0.01	<0.01	56.01	0.12	0.00
S20	2.12	0.54	5.18	*	/	0.22	0.01	0.01	47.97	0.23	0.01
S21	1.37	0.41	0.97	*	/	0.12	0.01	0.01	53.76	0.12	0.00
S22	1.71	0.60	0.54	*	/	0.19	0.01	0.02	53.57	0.13	0.01
S23	1.70	0.42	1.96	*	/	0.19	0.01	0.02	53.62	0.20	0.01
S24	1.80	0.63	0.62	*	/	0.18	0.01	0.02	53.35	0.13	0.01
S25	1.57	0.00	21.61	0.00		0.14	<0.01	0.01	30.13	0.11	<0.01
S26	2.84	0.02	20.98	*		0.10	<0.01	<0.01	30.23	0.16	<0.01
S27	1.70	0.16	6.86	0.03		0.14	0.01	<0.01	47.58	0.00	<0.01
S28	1.89	0.28	9.36	0.02		0.16	<0.01	0.01	44.05	0.10	<0.01

		content of partial trace element (ppm)				
sample no.	ignition loss (%)	Sr	Ba	U	Y	Sr/Ba
S1	/	325.42	96.99	1.11	6.29	3.36
S2	/	345.46	35.40	0.58	2.71	9.76
S3	/	200.07	42.58	0.76	5.88	4.70
S4	/	12.27	15.68	0.06	0.21	0.78
S5	/	55.35	11.48	0.09	0.51	4.82
S6	/	50.73	11.63	0.15	0.92	4.36
S7	/	20.66	3.79	0.04	0.13	5.45
S8	23.01	734.09	14.68	0.52	5.12	50.02
S9	17.56	723.66	263.11	1.16	8.94	2.75
S10	10.69	1187.78	58.67	1.36	7.82	20.25
S11	/	62.18	15.06	0.47	3.98	4.13
S12	/	80.92	49.30	1.07	7.19	1.64
S13	38.41	106.33	105.69	15.09	4.69	1.01
S14	25.10	343.08	143.76	1.84	8.29	2.39
S15	37.90	148.11	71.91	18.48	18.47	2.06
S16	41.10	76.91	13.30	0.47	3.59	5.78
S17	46.42	109.09	11.18	0.56	1.10	9.76
S18	44.61	58.21	13.21	1.34	1.10	4.41
S19	41.60	150.17	11.16	1.18	4.82	13.45
S20	42.88	95.47	15.69	4.86	3.51	6.09
S21	42.19	135.15	13.25	1.58	5.12	10.20
S22	42.18	132.16	15.49	2.35	4.89	8.53
S23	41.90	140.60	11.27	1.44	3.93	12.47
S24	42.23	139.85	15.80	2.70	4.77	8.85
S25	46.40	126.68	8.54	0.37	0.64	14.84
S26	45.63	152.87	14.10	0.26	0.55	10.84
S27	43.47	277.41	6.13	0.36	2.36	45.23
S28	44.10	181.15	9.69	1.01	0.89	18.70

a Remarks: (1) if the sample no. is consistent with Table 2, it represents the same sample source; (2) *: not detected; (3) /: unanalyzed.

What’s more, the rare earth element content in each rock type representing different stages of evaporation and concentration of Ordovician seawater in this area was analyzed systematically (see Table 2).

**Table 2:** Analytical Table of REE Content in the Ordovician Carbonate Rock-Gypsum/Salt Rock in the Eastern-Central Ordos Basin^a^

sample information		REE content (μg/g)														characteristic values and exception coefficient					
sample no.	lithology	La	Ce	Pr	Nd	Sm	Eu	Gd	Tb	Dy	Ho	Er	Tm	Yb	Lu	LREE	HREE	ΣREE	δEu	δCe	La′/Yb′
S1	brown halite rock	9.48	18.14	2.20	7.86	1.52	0.265	1.37	0.246	1.212	0.237	0.683	0.116	0.72	0.107	39.46	4.69	44.16	0.55	0.92	8.85
S2		4.79	9.24	1.22	4.49	0.77	0.124	0.71	0.109	0.528	0.103	0.297	0.053	0.31	0.045	20.63	2.15	22.79	0.50	0.90	10.37
S3		6.52	13.69	1.71	6.09	1.36	0.253	1.18	0.214	1.161	0.232	0.687	0.117	0.74	0.110	29.64	4.44	34.08	0.59	0.97	5.97
S4	white, grayish halite rock	0.32	0.59	0.08	0.28	0.05	0.011	0.05	0.008	0.040	0.008	0.024	0.003	0.02	0.003	1.34	0.15	1.50	0.68	0.87	9.52
S5		0.86	1.65	0.20	0.79	0.13	0.028	0.12	0.021	0.101	0.019	0.061	0.009	0.06	0.008	3.65	0.40	4.05	0.67	0.93	9.26
S6		1.40	2.58	0.32	1.21	0.21	0.038	0.18	0.034	0.166	0.035	0.106	0.017	0.12	0.016	5.75	0.67	6.43	0.58	0.90	8.14
S7		0.28	0.53	0.07	0.24	0.05	0.007	0.03	0.005	0.027	0.006	0.015	*	0.01	*	1.18	0.10	1.28	1.28	0.89	13.56
S8	anhydrite rock	6.54	17.59	2.77	10.40	1.55	0.225	1.17	0.190	0.912	0.172	0.477	0.072	0.44	0.066	39.08	3.49	42.57	0.49	0.99	10.07
S9		7.18	15.00	2.01	7.66	1.94	0.361	1.80	0.375	1.848	0.331	0.884	0.148	0.91	0.136	34.16	6.43	40.59	0.58	0.94	5.36
S10		11.65	21.09	2.51	9.12	1.81	0.327	1.50	0.295	1.566	0.311	0.926	0.162	1.04	0.154	46.50	5.96	52.46	0.59	0.90	7.57
S11	mud-powder crystal dolomite	3.96	8.45	1.04	3.82	0.82	0.183	0.77	0.142	0.729	0.144	0.404	0.065	0.41	0.061	18.27	2.72	20.99	0.69	0.98	6.57
S12		10.52	20.49	2.47	8.95	1.64	0.313	1.46	0.267	1.359	0.264	0.770	0.131	0.82	0.120	44.38	5.18	49.56	0.60	0.94	8.71
S13		1.67	3.77	0.58	2.44	0.69	0.155	0.64	0.165	1.013	0.194	0.546	0.089	0.51	0.076	9.31	3.23	12.54	0.70	0.92	2.21
S14		5.31	11.92	1.48	5.82	1.41	0.319	1.29	0.282	1.659	0.343	1.019	0.177	1.15	0.177	26.26	6.09	32.36	0.71	1.01	3.12
S15		2.26	5.92	0.90	3.83	1.21	0.271	1.36	0.414	3.091	0.744	2.527	0.47	3.15	0.457	14.38	12.21	26.60	0.64	1.00	0.48
S16		1.66	4.33	0.65	2.50	0.57	0.103	0.48	0.104	0.625	0.145	0.476	0.083	0.51	0.076	9.81	2.50	12.31	0.58	1.00	2.18
S17		0.61	1.49	0.24	1.01	0.25	0.038	0.21	0.043	0.224	0.041	0.118	0.018	0.10	0.015	3.63	0.77	4.39	0.49	0.95	4.10
S18		0.75	1.73	0.26	1.09	0.25	0.043	0.23	0.048	0.237	0.041	0.111	0.015	0.10	0.014	4.13	0.79	4.92	0.53	0.93	5.31
S19	limestone	13.58	25.68	3.29	11.74	2.10	0.289	1.58	0.237	1.012	0.175	0.429	0.057	0.32	0.047	56.69	3.85	60.54	0.46	0.90	29.14
S20		8.94	16.56	2.13	7.56	1.38	0.189	1.04	0.171	0.763	0.130	0.321	0.045	0.27	0.040	36.77	2.77	39.54	0.46	0.89	22.72
S21		14.66	27.47	3.53	12.60	2.22	0.311	1.68	0.261	1.102	0.188	0.437	0.059	0.33	0.046	60.79	4.11	64.90	0.47	0.89	29.75
S22		12.84	24.16	3.06	11.03	1.98	0.289	1.53	0.233	1.019	0.177	0.437	0.060	0.32	0.046	53.36	3.83	57.18	0.49	0.90	26.79
S23		14.81	26.87	3.20	10.71	1.79	0.233	1.37	0.205	0.849	0.145	0.346	0.05	0.29	0.043	57.61	3.29	60.90	0.44	0.90	34.75
S24		12.46	23.08	2.94	10.60	1.91	0.273	1.45	0.226	0.99	0.175	0.437	0.06	0.33	0.046	51.26	3.71	54.98	0.48	0.89	25.91
S25	powder-fine crystal dolomite	0.72	1.35	0.18	0.61	0.11	0.017	0.10	0.015	0.106	0.019	0.060	0.006	0.07	0.004	2.98	0.38	3.36	0.48	0.89	7.43
S26		0.44	0.86	0.12	0.45	0.10	0.015	0.08	0.010	0.083	0.013	0.049	0.002	0.05	0.002	1.97	0.29	2.26	0.53	0.89	6.14
S27	dolomitic spotted limestone	4.73	8.72	1.20	3.92	0.76	0.111	0.59	0.098	0.488	0.090	0.258	0.041	0.34	0.051	19.43	1.96	21.39	0.48	0.86	9.40
S28		1.81	2.88	0.35	1.16	0.21	0.032	0.19	0.026	0.150	0.026	0.078	0.007	0.08	0.006	6.44	0.55	7.00	0.49	0.82	16.24
detection limit		0.01	0.01	0.01	0.01	0.01	0.003	0.01	0.003	0.003	0.003	0.003	0.003	0.01	0.003						
standard values of chondrites (Boynton, 1984)		0.310	0.808	0.122	0.600	0.195	0.074	0.259	0.047	0.322	0.072	0.210	0.032	0.209	0.033	2.1	1.18	3.3	1.00	1.00	1.00

a Remarks: (1) if the sample no. is consistent with the one in Table 1, it represents the same sample source; (2) La′/Yb′: the ratio of lanthanum and ytterbium element chondrites after standardization; (3) *: none detected.

### 3.2 Basic Situation of the Instruments and Equipment Used

#### 3.2.1 Main Element Analysis

The major elements were determined by means of an X-ray fluorescence spectrometer, whose instrument model is Shimadzu XRF-1800.

The standard substances used are GSR2, GSR5; the elemental measurement range: O–U; accuracy: ± 0.6%.

##### 3.2.1.1 Sample Treatment

The sample was crushed to a particle size of less than 75 μm (200 DPI), then dried in an oven at 105 °C for 2–4 h, and after removal, it was placed in a dryer to cool to room temperature, and then it was entered the analytical test process.

##### 3.2.1.2 Experimental Test Method

First use lithium tetraborate to melt, use ammonium nitrate as an oxidant, and add lithium fluoride and a small amount of bromide as a flux and a demold agent. The mass ratio of the sample to flux is 1:8. It is melted on a melting sample machine at 1150–1250 °C to make a glass sample. Measurements are taken on an X-ray fluorescence spectrometer, and the composition is calculated according to the fluorescence intensity.

#### 3.2.2 Trace Element Analysis

The trace elements were determined by using an inductively coupled plasma mass spectrometer, whose instrument model is Thermo Fisher iCAP RQ.

The standard substances used are GSR2, GSR5; resolution (mass resolution per unit, amu) ≤0.8.

##### 3.2.2.1 Sample Treatment

The sample was crushed to a particle size of less than 75 μm, placed in an oven at 05 °C for 2–3 h, and then placed in a dryer to cool to room temperature.

##### 3.2.2.2 Experimental Test Method

50 mg of the sample was accurately weighed below 200 meshes and prepared into a test solution for direct determination by fluoric acid and nitric acid digestion, using the ICP-MS external standard method. The content of trace elements was calculated by standard curve calibration.

#### 3.2.3 Rare Earth Element Analysis

The rare earth elements were determined by using an inductively coupled plasma mass spectrometer, whose instrument model is PerkinElmer NexION 350.

The standard substances used are GSR2, GSR5; resolution (mass resolution per unit, amu) ≤ 0.8.

##### 3.2.3.1 Sample Treatment

The sample was crushed to a particle size of less than 75 μm (200 DPI), placed in an oven at 05 °C for 2–3 h, and then placed in a dryer to cool to room temperature.

##### 3.2.3.2 Experimental Test Method

50 mg of the sample was accurately weighed below 200 meshes and prepared into a test solution for direct determination by fluoric acid and nitric acid digestion, using the ICP-MS external standard method. The content of rare earth elements was calculated by a standard curve calibration.

#### 3.2.4 Scanning Electron Microscopy Analysis

SEM (scanning electron microscopy) observation and analysis were achieved with the help of a field emission scanning electron microscope, whose instrument model is ZEISS Sigma 300. Gold particle standard material: TED PELLA No.617; Magnification: 10–1,000,000×.

##### 3.2.4.1 Sample Treatment

First, removal of oily contaminants from specimens according to the standard regulations. When the sample is piled up, the observation surface should be fresh and flat and close to the bedding plane. The thickness of the sample should not be greater than 5 mm. Then, the sample surface is plated with gold (5–20 nm) and becomes the analysis target sample.

##### 3.2.4.2 Experimental Test Method

The analyzed target sample is sent into the sample chamber of the scanning electron microscope, and then the vacuum is pumped. The sample is scanned by a focused electron beam, which excites secondary electrons on the surface of the rock sample. The secondary electrons are collected by the detector, converted into optical signals by the scintillator, and then converted into electrical signals by the photomultiplier tube and amplifier to control and adjust the intensity of the electron beam on the fluorescent screen and display the scanning image synchronized with the electron beam, reflecting the surface morphology of the sample.

#### 3.2.5 Energy Spectrum Analysis

The energy spectrum analysis whose instrument model is Bruker Quantax XFlash 6–30.

##### 3.2.5.1 Standard Material of Gold Particles

TED PELLA No.617; Elemental detection range: Be ∼ Am; Energy resolution: Mn Ka-123 eV, C Ka-45 eV, and F Ka-53 eV.

##### 3.2.5.2 Sample Treatment

Energy spectrum analysis is an analytical device matched with a scanning electron microscope, and its sample treatment is the same as that of the scanning electron microscope.

##### 3.2.5.3 Experimental Test Method

The high-energy focused electron beam of the scanning electron microscope bombards the surface of the sample, causing the various elements on the surface of the sample to be subjected to different characteristic X-rays. The position and intensity of the energy spectrum peaks of the characteristic X-rays can be used to determine the elements contained in the sample and their contents. The ratio of the intensity value of a certain element’s spectrum peak in the sample to the intensity value of the spectrum peak of the standard substance is an approximation of the content of that element. After the model of the ZAF method is corrected, the content value of the element in the sample can be obtained.

### 3.3 Laboratory’s Analytical Qualifications

All of the above analyses were completed by Sichuan Keyuan Testing Center of Engineering Technology Co., Ltd., whose laboratory has passed China Inspection Body and Laboratory Mandatory Approval (CMA 242301061196) and China National Accreditation Service for Conformity Assessment (CNAS L6561).

## 4 Results

### 4.1 Main Rock Composition

The Ordovician carbonate rock and gypsum salt rock system in this area is mainly composed of limestone, dolomite, anhydrite rock, and salt rock (part of transitional dolomite limestone, gypsum dolomite, and so on, can also be developed in some intervals), and the typical lithology of the main rock types and the distribution and development of the characteristics of the main rocks are briefly described as follows:

#### 4.1.1 Limestone

It was formed in a normal marine sedimentary environment during the transgressive sedimentation period. The limestone is mainly composed of calcite and mostly appears in the form of thick layers. It is mainly distributed in the Ma 4, Ma 5, Ma 6, and Ma 2 Members in the eastern part of the basin (Figure 5). The lithology is mainly micrite, marl, and grainstone, which is mainly composed of fossil fragments of ostracods, gastropods, and others, with porphyritic dolomitic structures in some parts (Figure 6A–C).

**Figure 6:**
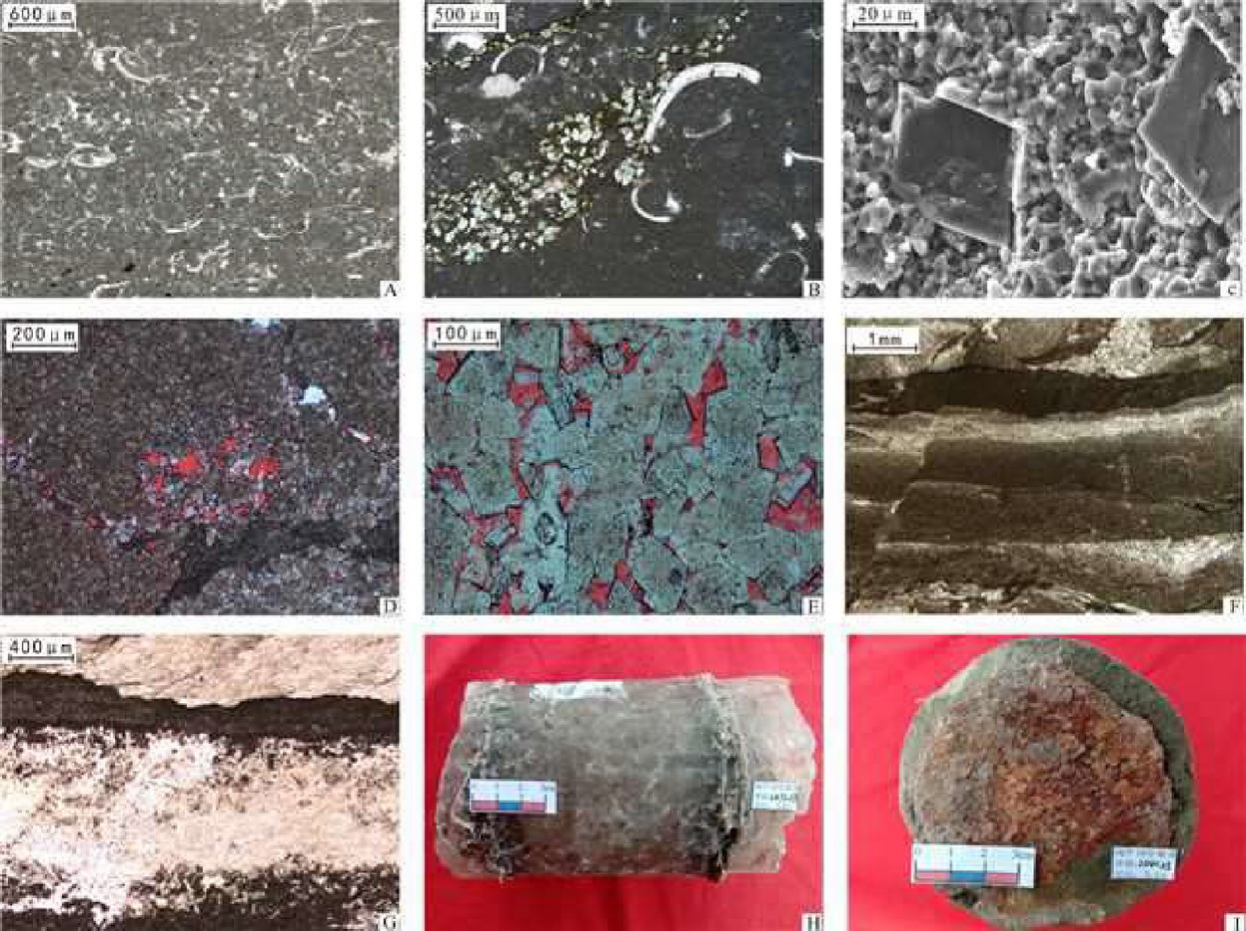
Structural characteristics of the main rocks in the Ordovician C-G-S rock system in the Ordos Basin. (A) Well Y48, Ma 5^5^, 2767.18 m, bioclastic micrite spherulitic limestone, with spary and microsparkling cementation locally, plane polarized light; (B) Ma 4 Member of Well GP1, 2663.35 m, containing bioclastic micrite, local porphyritic dolomitization, plane polarized light; (C) Ma 4 Member of Well GP1, 2642.0 m, dolomite-bearing micrite, matrix of micrite calcite, compact structure, large authimorphic rhombohedral grains dolomite, scanning electron microscope; (D) Well T18, Ma 5^5^, 3702.12 m, powder crystal dolomite, the red spot indicates the pore mold, plane polarized light; (E) Ma 4 Member of Well E9,3859.60 m, fine crystalline dolomite, dolomite fine-grained grain structure, highly euhedral, intercrystalline pores (red pore mold), plane polarized light; (F) Well S473, Ma 5^6^, 3767.01 m, gypsum-bearing mud and crystalline dolomite, with dislocation structure from dehydration, plane polarized light; (G) Well ZJ1, Ma 5^10^, 2597.26 m. Thin layer of anhydrite rock intercalated with mud crystal dolomite, fine-grained anhydrite structure, snowflake-like structure, plane polarized light; (H) GP1, Ma 5^6^, 2473.28 m, grayish, white rock salt rock, coarse crystal granular structure, particle size 1–2 cm, thin layer of dolomite, core photograph; (I) Well GP1, Ma 5^6^, 2441.25 m, brown-red halite rock, medium-coarse crystalline granular structure, particle size 0.3–0.6 cm, core photograph.

#### 4.1.2 Dolomite

This type of rock is distributed on a certain scale in both the transgression and regression periods and is mainly composed of dolomite. The formation of dolomite is controlled by dolomitization caused by seawater salinization and gypsum precipitation. The dolomite is evidently characterized by local and stratiform distribution (Figure 5). The dolomites in the Ma 4, Ma 5, and Ma 2 Members precipitated during the transgression period are mostly dolomite with powder crystal or fine crystal structure (Figure 6D,E), generally in the form of medium-thick or large-sized dolomites. The dolomites in the Ma 1, Ma 3, and Ma 5 Members deposited during the regression period are mostly dolomites with a mud-powder crystal structure, often in thin layers or interbedded with anhydrite, showing relatively evident lamellar structure (Figure 6F).

#### 4.1.3 Anhydrite Rock

It was mainly formed in the early stage of the high-standing-regressive system tract, composed mainly of anhydrite minerals. The increase in the degree of limitation of the depression basin led to the precipitation of gypsum (or anhydrite) minerals, which were mainly developed in the regressive sedimentation sequence of Ma 5 and Ma 3 Members. Geographically, the distribution was more developed near the edge of the salt depression basin, mostly in the form of interactive development with medium-thick layers and dolomite layers. The lithology is dominated by white anhydrite, of which the anhydrite crystals are mostly fibrous, fine-grained structures. The rocks are mostly snowflake-like structures with dolomitic bands or thin layers in some parts (Figure 6G).

#### 4.1.4 Halite Rock

It was mainly formed in the regression sedimentary period, and it is mainly composed of halite, with occasionally potassium halite and other end-evaporating minerals. At this time, because the salt depression basin was basically isolated from the open sea, it entered the middle and late stages of drying and evaporation. The seawater was highly concentrated, and the crystallization and precipitation of halite started. The halite rocks in this area were mainly developed in the regression sedimentary sequence of the Ma 1, Ma 3, and Ma 5 Members. The location of halite was generally limited to the sedimentary area of the salt depression basin in Northern Shaanxi, and most of them are concentrated and developed in large intervals of thick layers. There is a thin layer of dolomitic intercalation locally. Most of the rocks have a pure and transparent coarse-giant crystal grain structure, and some of the layers are gray-white, grayish, or light brown-red (Figure 6H,I).

### 4.2 Formation Stage of the C-G-S Rock System

Janecke first proposed the concept of continuous sedimentary facies of seawater evaporation and the four-stage evaporation model: (I) limestone and dolomite; (II) gypsum; (III) halite (+gypsum); (IV) Na–Mg sulfate, and then potassium salt.^88^ The mineral concentration and remaining volume of seawater in each stage are also given. However, due to the sealing of the evaporation basin, sea level fluctuations, and other factors in the actual depositional process, the development of the real strata is more complicated, especially in the cyclic sedimentary sequence. Most of the evaporation cycles have not yet reached the final stage of the evaporation cycle as a result of interruption from a new round of sea level rise caused by external seawater injection. It is naturally no exception in the formation of Ordovician evaporites in the Ordos Basin. Therefore, based on the development characteristics of Ordovician carbonate rocks and evaporative gypsum and salt rocks in this area, the chemical precipitation process in marine sediments is divided into the following four main stages (Table 3).

**Table 3:** Brief Table of the Rock-Forming Stages of the C-G-S System of Ordovician in Central-Eastern Ordos Basin

rock-forming stages	sea level change situation	the closed condition of the water body	seawater concentration level	the main mineral crystallized out	typical rock types
early stage	marine transgression period	connect to the open sea	normal seawater salinity (salinity 3.5%)	precipitation of calcite (aragonite)	limestone,mud-powder crystal, dolomite
middle stage	high-water period	semiconfined	concentrated to more than 5 times the normal salinity of seawater (salinity >15%)	precipitation of gypsum and dolomitization	anhydrite rock, local limestone segments are dolomitized
late stage	low-water period	basically closed	the brine concentration more than 10 times the normal salinity of seawater (salinity >26%)	halite precipitation	white, grayish halite rock
final stage	late low water	completely cut off from the open sea	the brine concentration reaches 30 times the normal salinity of seawater (salinity >33%)	potassium halite precipitation	potassium halite rock

#### 4.2.1 Early Stage: Precipitation of Calcite (Aragonite)

When the seawater was slightly concentrated, the least soluble carbonates (mainly calcium carbonate) began to precipitate. This is also the reason for the carbonate precipitation that is occurring in the modern tropical marine environment, which is also a result of the comprehensive involvement of biochemical effects. Micrite, limestone with a bioclastic-granular mud structure, was mainly formed in this area. Granular shoal limestone deposits were also locally developed in the shallow water environment, but there was mostly the formation of dolomite due to dolomitization.

#### 4.2.2 Middle Stage: Precipitation of Gypsum and Dolomitization

When the seawater further evaporates, and the salinity reaches 15–17%, gypsum minerals begin to precipitate. At this time, the salt depression basin of northern Shaanxi is highly isolated from the open sea, and the convective circulation of seawater is evidently restricted, mainly resulting in the formation of anhydrite rock and gypsum dolomite sedimentary layers.

In addition, recent studies have shown that most of the dolomitization in the carbonate-gypsum-salt rock system in this area was related to the precipitation of gypsum matter. That is, the precipitation of CaSO_4_ caused the increase of the Mg/Ca ratio, thereby resulting in strong dolomitization of the early lime sediments (in a shallow-buried diagenetic environment) and forming a large-scale dolomite strata in this area.

#### 4.2.3 Late Stage: Halite Precipitation

When the mineralization degree of seawater reaches 26%, halite minerals begin to crystallize. The Northern Shaanxi Salt Depression Basin in the Ordovician reached this evaporation stage in the Ma 1, Ma 3, and Ma 5 Members. At this time, the Northern Shaanxi Salt Depression Basin was blocked by the Central paleo-uplift, the Lvliang paleo-uplift, and other surrounding uplifts. The open sea was almost completely isolated, and it had entered a stage of strong evaporation and concentration, forming thick and pure halite layers.^79,86^

#### 4.2.4 Final Stage: Potassium Halite Precipitation

When the climate was extremely dry and the seawater was evaporated and concentrated to a salinity of 33% (density of 1.31), potassium salt began to crystallize. A large-scale potassium halite layer has not yet been found in the Ordovician in this area. But in the brown-red halite layer in the Ma 5^6^ Submember of the salt mine exploration well in the Suide area in the east of the Ordos basin, a thin layer of halite with a higher potassium content has been found, with K content reaching up to 4.92%.^89^ Potassium salt crystals are mostly irregular granular; they are elongated granular, followed by euhedral and semieuhedral cubes, which are closely symbiotic with halite. Potassium minerals are mainly distributed in small pores between the halite crystal grains, and sometimes their fine crystals are enveloped by the halite. In addition, it was also found that there is potassium iron salt, a small amount of carnallite, and other salt minerals at the end of evaporation in the symbiotic minerals, indicating that some halite layers in this area have indeed entered the final stage of drying evaporation, but no large-scale enrichment of potassium has been found yet. However, there is still the potential of forming thicker potassium deposits in local depressions.^90,91^

### 4.3 Major and Trace Elements

Table 1 shows the complete chemical analysis of major elements and the results of the inductively coupled plasma mass spectrometry (ICP-MS) analysis of some trace elements, such as Sr and Ba, of the representative rock samples selected for this experiment. With the analysis of mineral composition of the carbonate-gypsum-salt rock system in this area by scanning electron microscopy, etc., it can be seen that NaCl, CaO, and MgO in the table basically represent the elemental compositions of autogenous minerals such as halite, anhydrite, calcite, and dolomite in the rock samples. The Al_2_O_3_ and SiO_2_ represent mainly the compositions of terrigenous clastic minerals such as Illite, feldspar, and quartz, and a small amount of authigenic quartz.

It can be seen from Table 1 that for the grayish and white halite rock, the halite mineral accounts substantially for its high purity. The content of NaCl is more than 90%, and some are as high as 98%, only with a small amount of clay and authigenic quartz, and the like. The brown-red halite rock is compositionally mixed. In addition to the main authigenic mineral of halite, it still contains a certain amount of dolomite, and terrigenous clastic minerals such as Illite, quartz, feldspar, and others. Therefore, the NaCl content is generally above 50–70%, while the contents of MgO, Al_2_O_3_, and SiO_2_ are evidently higher, reaching 1.5–4, 1–3, and 4–10% respectively. And potassium (K) content in brown-red halite rock is relatively high, reaching 0.4–0.7%, which is 3–5 times higher than that in white halite rock.

In anhydrite rock, in addition to the absolute predominance of anhydrite, it contains a certain amount of dolomite and terrigenous clastic minerals. As a result, the content of CaO in its elemental analysis is more than 50%. The contents of MgO, Al_2_O_3_, and SiO_2_ are higher than those of the grayish and white halite rock but closer to those of the brown-red halite rock.

As for limestone and dolomite, their lithologies are relatively pure. Among them, calcite and dolomite minerals are absolutely dominant, and the content of clay minerals is low, resulting in Al_2_O_3_ content below 0.5%, but the rock often contains a certain amount of authigenic quartz, so its SiO_2_ content is generally 1–4%, and can reach more than 5% individually.

In addition, the overall analysis results of various types of rocks show that the Fe content in rocks is generally low, and the content of total iron (TFe) is generally below 1%. The Fe content of brown-red halite is higher than that of white halite. The Fe content of anhydrite and a few dolomites is slightly higher than that of other rocks. However, the contents of Mn, Ti, P, and other elements are generally extremely low. The MnO content of most samples is only 0.01% or less than 0.01%, and the TiO_2_ content is mostly below 0.1%. That of P_2_O_5_ is mostly below 0.05%.

Sr and Ba, which are usually trace elements, have relatively high contents in the evaporite series in this area, especially in anhydrite rock. The content of Sr in anhydrite rock is significantly higher than that in other rocks, up to more than 700 ppm (with a maximum of 1187.78 ppm). The contents of Sr in other rocks are mostly below 200 ppm. However, the Sr content of the brown-red halite rock is also relatively high (some can be as high as 300 ppm), which is significantly higher than the Sr content in the white pure halite. The content of Ba is also significantly higher in anhydrite rocks and brownish-red halite rocks, mostly above 30 ppm (up to 263.11 ppm) and below 15 ppm for other rocks.

Among the relatively low content of U, Y, and other elements, different rock types also show a certain trend of change. For example, in addition to the extremely low content of U element in pure white halite rock (<0.2 ppm), its content in other rocks is generally 1–5 ppm. The content of Y and U elements shows evident similarities, which are also extremely low in the white salt rock (<1 ppm), but slightly higher in other rocks, generally in the range of 1–10 ppm.

### 4.4 Rare Earth Element in Different Rocks

Through systematic sampling and analysis of the rare earth element of different rocks in the Ordovician carbonate-gypsum-salt rock symbiotic system, it is shown that due to the differences in the mineral compositions of different rocks, and in the water medium conditions during mineral precipitation (such as salinity of seawater), there will be great differences in the content and distribution mode of the rare earth element for each rock type (Table 2).

#### 4.4.1 REE in Carbonate Rocks (Limestone, Dolomite)

##### 4.4.1.1 Limestone

The REE content of the limestone of the Ma 5^5^ Submember is generally higher than that of halite rocks. The total REE content is more than 30 ppm, and the REE content of limestone is higher than that of dolomitic porphyry limestone, evidently showing the enrichment of light rare earth elements in the distribution (Figure 7). The La′/Yb′ ratio (the ratio of the chondrite-normalized value of La and Yb) is more than 20. The samples show evident negative Eu anomalies, and the abnormal value of Eu (δEu) is mainly distributed between 0.45 and 0.50.

**Figure 7:**
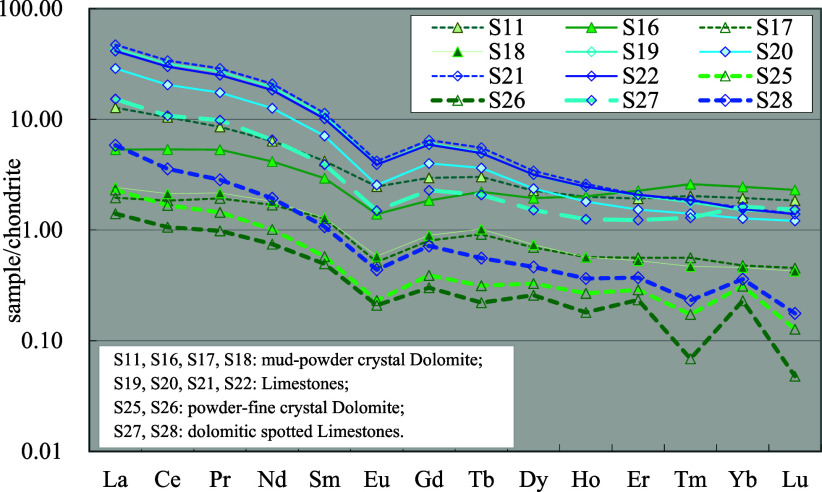
Distribution model of rare earth elements in limestone and dolomite (see Tables 1 and 2 for other sample information).

In the dolomitic limestone of the Ma 4 Member, the total rare earth elements and the abnormal amplitude of light/heavy rare earth elements are significantly reduced. With the increase of dolomite content, the total rare earth elements also gradually decrease, and some of the characteristics of the dolomite with granular structure in the Ma 4 Member appear, such as the odd-to-even predominance of heavy rare earth elements at the end.

##### 4.4.1.2 Dolomite

compared with limestone, the REEs of the dolomites of the Ma 5^6^ Submember are significantly reduced. The total content of rare earth elements mostly ranges from 10 to 30 ppm and the distribution of rare earth elements shows evident differences between light and heavy rare earth elements. The La′/Yb′ ratio is mostly in the range of 2–10 and the characteristics of negative Eu anomaly are similar to limestone but with a slight reduction. The heavy rare earth elements tend to be flat on the distribution curve, and some samples show a slightly higher tendency to the right (Figure 7).

The dolomite with granular structure of the Ma 4 Member has a significantly lower content of rare earth elements and the total content is mostly below 10 ppm, which is generally lower than that of dolomite in the Ma 5^6^ Submember. The differences between light and heavy rare earth elements are also more distinct than those in the Ma 5^6^ Submember, with the La′/Yb′ ratio mostly being between 6 and 12. There is evident odd-to-even predominance in heavy rare earth elements.

#### 4.4.2 REE in Evaporative Gypsum-Salt Rocks (Anhydrite, Halite Rocks)

##### 4.4.2.1 Anhydrite Rock

The rare earth element content of anhydrite rock is similar to that of limestone and has a tendency to be higher generally. The total content of rare earth elements is mostly in the range of 40–50 ppm, but the differentiation of light and heavy rare earth elements is significantly lower than that of limestone (Figure 8). The La′/Yb′ ratio is mostly in the range of 5–10, and there is still a negative Eu anomaly, but it slightly decreases. The δEu values are mainly distributed in the range of 0.50–0.60.

**Figure 8:**
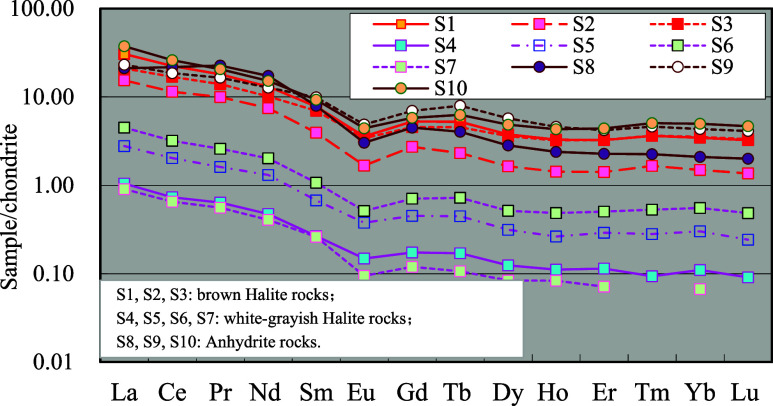
Distribution model of rare earth elements in anhydrite and halite (see Tables 1 and 2 for other sample information).

##### 4.4.2.2 Halite Rock

Pure halite rocks (white and gray halite in Table 2) contain an extremely small number of rare earth elements, and the total content of rare earth elements is less than 2 ppm. Almost all the chondrite-normalized value of rare earth elements are within 1.0–0.1 or even lower. The distribution curve of REE is still characterized by the enrichment of light rare earth elements (Figure 8), and there are also some negative Eu anomalies, which are mainly distributed between 0.60 and 0.70, but the distribution morphology of heavy rare earth elements is gradually flattening out.

As for the light brown and brick-red halite rock with high impurity content and a small amount of potassium, its rare earth element content and distribution characteristics are completely different from those of pure halite rock and are different from those of mud crystalline dolomite and anhydrite in the Ma 5^6^ Submember. The rare earth element distribution characteristics of gypsum rock are relatively close. The total content of rare earth elements is mostly 5–20 ppm, showing the characteristic of enrichment of light rare earth elements. The La′/Yb′ ratio is 9–12 and the distribution curve of heavy rare earth elements also has a tendency to be flat.

### 4.5 Variation Trend of REE in C-G-S Rock Evolution

The carbonate-gypsum-salt rock sequence is a sedimentary system of endogenous origin caused by chemical or biochemical precipitation. It is composed of limestone-dolomite-anhydrite-halite-potassium salt, which basically represents the evaporation and concentration of seawater or even the process of complete drying evaporation. In order to analyze the varying characteristics of the geochemical behaviors of rare earth elements in the process of evaporation, concentration, and deposition, the rare earth element analysis results of representative rock samples at different deposition stages were displayed in the same rare earth element distribution curve diagram to intuitively compare the content of rare earth elements in the evaporation and concentration process and the variation characteristics of distribution curve morphology. The following important trend changes can be mainly reflected in Figure 9.

**Figure 9:**
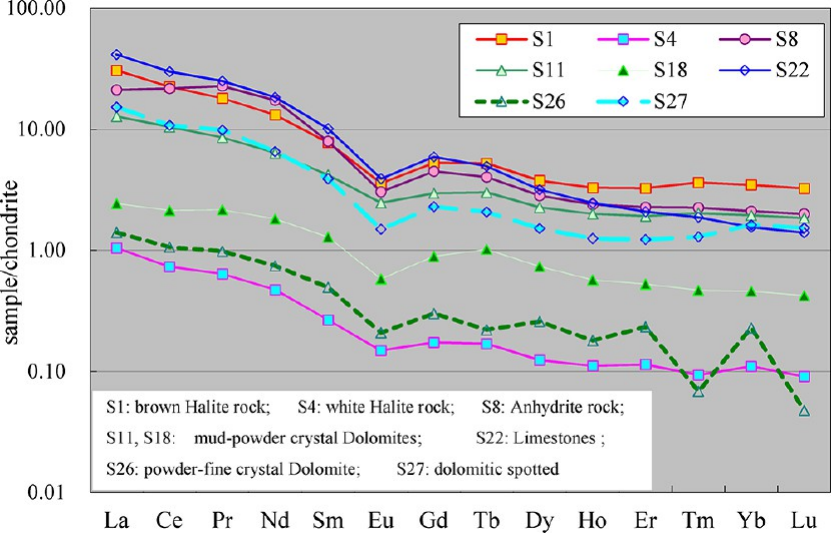
Comparison of rare earth element distribution patterns in the limestone-dolomite- gypsum-salt evolution sequence of the Majiagou Formation (see Tables 1 and 2 for other detailed sample information).

#### 4.5.1 All Rocks in C-G-S Have a Similar REE Distribution Pattern

The limestone, dolomite, anhydrite rock, and halite rock in the carbonate-gypsum-salt rock symbiotic system are all basically similar in the morphology of the REEs distribution curve. That is, the rocks are all rich in light rare earth elements and have a certain negative Eu anomaly. There is basically no evident Ce anomaly, but the content of rare earth elements varies greatly among different types of rocks.

#### 4.5.2 REE Content in Limestone and Anhydrite Is Higher Than That in Halite

As shown in Figure 9, the chondrite-normalized values of the rare earth elements in limestone and anhydrite rocks are more than 1, and some light rare earth elements are above 10. However, the chondrite-normalized values of rare earth elements of pure halite rocks are mostly below 1; only the first light rare earth element La of individual samples is slightly higher than 1, mostly the previous part is higher than 1.0, and the latter is less than 1.0 for light rare earth elements. While the chondrite-normalized value of rare earth elements in dolomite is generally between the above two.

#### 4.5.3 Decreasing of the REE Content from Limestone to Dolomite

It can be seen from the figure that in carbonate rocks, the content of rare earth elements in limestone (shown by curve No. S22) is significantly higher than that of dolomite (shown by curve No. S11, S18, and S26), by an order of magnitude. However, the distribution curves of the two are still relatively close in morphology. The rare earth elements content of porphyry limestone (shown in the curve of Figure 9, No. S22) is between the two. However, it is worth noting that in the dolomites of the Ma 4 Member, the heterogeneous characteristics of the odd-to-even predominance of heavy rare earth elements begin to become prominent, which is manifested in the process of dolomitization (basically synchronous with the precipitation of gypsum) from limestone to dolomite. In addition to the replacement of the constant Mg element, the rare earth elements also experienced evident migration.

#### 4.5.4 Increasing of the REE Content in the Final Stage of Evaporation

In the process of stepwise crystallization evolution from limestone-dolomite-anhydrite-halite rock, the content of rare earth elements in various types of rocks showed a decreasing trend. However, strangely, the content of rare earth elements has increased significantly in the light brown-red halite rock (as shown by the curve in Figure 9, No. S1), which was formed in the latest stage of halite crystal precipitation. For this phenomenon, drying evaporation should be the most reasonable explanation for its cause.

As mentioned above, in the final stage of the “drying evaporation” of the salt depression basin, potassium halite will be formed, and the rare earth elements in the seawater will eventually be deposited in the latest sediments as the salt depression basin dries. This will lead to the final formation of potassium-rich halite deposits, which should have the highest content of rare earth elements. However, the practical situation is still somewhat complicated, which is mainly manifested in the fact that the salt minerals that are finally precipitated do not appear in separate layers in most cases but are evenly dispersed and precipitated in the salt crystals of the earlier halite deposits. In the intergranular pores, only when there are late synsedimentary depressions locally, and a large amount of concentrated brine converges in a smaller area, will the most advanced evaporative salt minerals, such as potassium and other highly enriched potassium, appear. Halite layer^92^ is also the main reason for the harsh mineralization conditions of the sedimentary potassium salt deposits and the difficulty in finding the ore layers.

Therefore, the light brown-red halite rock formations that have been discovered in this area have relatively high potassium content, with the highest potassium content reaching 4.92%,^89^ which basically represents the final “drying evaporation” of some salt depression basins. Although the rare earth element content has increased to a certain extent, it has not yet reached a high degree of enrichment. This is precisely because the evaporation residual minerals precipitated in the latest stage cannot be enriched in separate layers but are dispersed in the intercrystalline pores as a result of early crystallization of minerals in halite rocks.

The overall shape of the REEs distribution curve is extremely similar to that of the granite in the Sanjiang area of western Sichuan-East Tibet and the 430 granite body in the Xianghualing area of Hunan,^93^ indicating that its terrigenous clastic sediments may be sourced from the granite provenance or strongly influenced by the acidic volcanic debris in the adjacent area. In addition, the anomaly of the yttrium element usually represents a strong volcanic eruption,^94^ and evident Y anomalies are generally seen in the source rocks of this area, which also reflects that it may be affected by the addition of strong neutral-acid volcanic debris.

## 5 Discussion

### 5.1 Negative Eu Anomalies May Reflect a Depositional Environment of Peroxidation

It can be seen from the above analysis (see Figures 7, 8, and 9) that in the C-G-S rock system, the rare earth element distribution curves of the main rock type (such as limestone, dolomite, anhydrite, and salt rock) all have evident negative Eu anomalies. This is especially true for carbonate and sulfate minerals. Only the Eu anomalies in pure halite rocks are slightly weak.

Although rare earth elements have great similarities in atomic structure and chemical properties, the processes and forms of geological action in nature are extremely different, resulting in a certain degree of fractionation of rare earth elements in their different geological processes. According to previous studies, the main factors that affect the fractionation of rare earth elements in nature are attributed to differences in crystal chemistry, element alkalinity, environmental oxygen fugacity, stability of complex formation, and ion adsorption capabilities.^95^ For carbonate and sulfate minerals with divalent Ca^2+^ and Mg^2+^ as metal cations, and CO_3_^2–^ and SO_4_^2–^ as anions, the divalent rare earth elements such as Eu, Sm, and Nd, are most likely to enter the crystal lattice of calcite, dolomite, and anhydrite minerals in the form of isomorphism to produce the differentiation of rare earth elements if there is appropriate ambient oxygen fugacity. At least, a positive anomaly of Eu should appear. However, the practical situation in this area is just the opposite. In limestone, dolomite, and anhydrite, the REEs all appear as a negative Eu anomaly, which indicates that the rare earth elements do not enter into the crystal lattice of carbonate and sulfate minerals as the main existence form. It also reflects that the oxygen fugacity of the deposition medium environment at that time may still be in a partial oxidation state.

Ce in the C-G-S system of this area basically shows no anomaly, or shows a weak negative Ce anomaly (δCe is at 0.8–0.9) in the formation of relatively deep water limestone, fine-grained dolomite, which also reflects a slightly oxidizing depositional environment as a whole. In addition, Fe, which is more sensitive to the comparison of oxidation–reduction, also has a more obvious abnormal response in different rock types, such as the total iron (TFe) in extremely oxidized anhydrite and tidal flat mud powder crystalline dolomite are usually between 0.5 and 1.2%, while the total iron content in slightly oxidized limestone and fine grained dolomite are usually <0.25%. Therefore, it can be inferred that the C-G-S system in this area was mainly formed under an oxidizing depositional environment, based on the comprehensive analysis of Eu anomaly, Ce anomaly, and sensitive elements such as Fe.

This is consistent with the geological fact that the oxidizing sulfate mineral anhydrite (CaSO_4_) is present in the C-G-S rock system in this area, but the reducing sulfide mineral pyrite (FeS_2_) is not found. And it can explain to some extent why the biological productivity when rock forming in this area is high, while the organic matter remaining in the rocks is very low.

There are literature reports that the Archaean carbonate rocks in India formed in marine environments have obvious positive anomalies in Eu,^96^ and the carbonates of Late Neoproterozoic successions in India have obvious negative anomalies in Eu.^97^ If we zoom in on the time scale, does it indicate that the Great Oxygenation Event (GOE), which occurred approximately 2.4 billion to 2 billion years ago,^98–101^ and the Eu anomaly in carbonate sediments, have a globally consistent prevalence?

### 5.2 Clay Minerals Are Not a Major Source of REE in C-G-S

The REEs are minor constituents of seawater in modern oceans, having concentrations of only a few nanograms per liter.^102,103^ They show certain differences among different oceans and have a significant increasing trend with the increase of seawater depth.^104,105^ However, deep-sea soft mud typically has a high content of rare earth elements. For example, in the deep-sea mud of the Pacific Ocean, the REE and Y (REY) rich mud (generally metalliferous sediment, zeolitic clay, and pelagic red clay in lithology) has high REY contents up to 1000–2230 ppm, even as a potential resource for REE.^106^

Some studies have suggested that the adsorption of rare earth elements by clays may be an important pathway for the precipitation of rare earth elements from seawater.^107^ However, this may not be the case through the analysis of the C-G-S rock system in the area of Ordos area. The results are mainly manifested in the following two aspects: One is that most of the carbonate-gypsum-salt rock system in this area is relatively pure in lithology, with a low content of clay minerals, which is generally less than 3%. The high contents of clay minerals in some samples are only 5–10%. The clay minerals may have a certain adsorption effect on rare earth elements but do not have much influence on the overall rare earth element content of the rock. Second, the correlation analysis between the content of rare earth elements in various rocks and that of clay minerals (represented by the content of Al_2_O_3_ in the analysis of major elements) shows that there is no evident correlation between them (Figure 10, red dot series), with correlation coefficient R^2^ of only 0.14, indicating that the adsorption of clay minerals is not a decisive factor influencing the content of rare earth elements in the carbonate-gypsum-salt rock system in this area.

**Figure 10:**
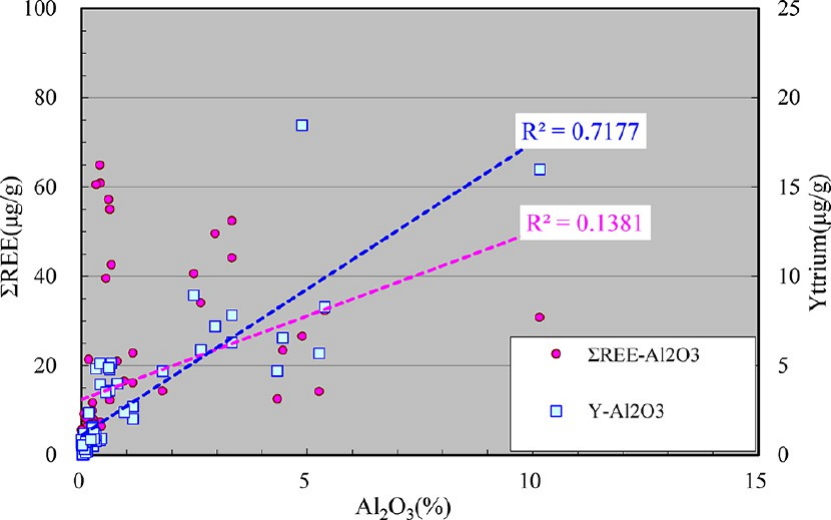
Scatter diagram of total REEs and yttrium content with clay content in the Ordovician carbonate-gypsum-rock of the Ordos Basin

In addition, the correlation analysis shows that the content of the yttrium element (Y) in various rocks in this area is closely related to the content of clay minerals. The correlation coefficient *R* ^2^ between the content of Y and that of clay minerals represented by Al_2_O_3_ reaches 0.72 (Figure 10, blue square series). The anomaly of the yttrium element usually represents a strong volcanic eruption.^94^ Thin altered tuff interlayers are generally seen in the carbonate-gypsum-salt rock system in this area, which also reflects the strong influence of pyroclastic materials in the short term under the sedimentary background of carbonate and evaporative gypsum-salt rock. It also shows that the small amount of clay mineral impurities dispersed in carbonate rocks and gypsum-salt rocks that are not stratiform may have mainly originated from the atmospheric fall of remote volcanic eruptions. During the depositional period of the Ordovician Majiagou Formation, the Ordos area and even the North China block are basically located in the epicontinental marine sedimentary area. The adjacent continental area that could provide terrigenous clastic material was extremely small and mainly limited to the Yimen uplift area, where the supply of terrigenous clastic material was relatively limited. Therefore, the tephra falling from the sky may be the main source of clay impurities in the strata.

The clay minerals in the C-G-S system of this area mainly originate from volcanic ash and can be further corroborated from several aspects in addition to the aforementioned Y anomaly: the first one is that the rocks rich in clay minerals generally have an abnormal U element, which is basically positively correlated with the aforementioned Y element anomaly; the second is that the layers with concentrated mud usually have a very fine volcanic debris structure and are identified as tuff layers; the third is that during the deposition period of the Majiagou Formation which formed the system, the Ordos and even the North China Block were basically located in the marine depositional environment, lacking the erosion ancient land that could provide the soil-derived debris material, except for the Yimen ancient land at the northernmost edge.

### 5.3 Main Forms of REE’s Existence in the C-G-S Rock System

The absolute and relative concentrations of the REEs in ocean waters reflect their input from rivers, by aeolian transport, and from hydrothermal vents; their interaction with the biogeochemical cycle involving removal from surface waters by adsorption and oxidation at particle surfaces (probably with organic coatings) and deeper regeneration, and the effects of advective transport.

The rare earth elements in marine sediments mainly come from the injection of terrestrial rivers, or sediments falling from the atmosphere (especially volcanic ash), submarine hydrothermal vents, and volcanic eruptions.^105–110^ Whether it is in the river water from land into the sea, or in the seawater, the main forms of REEs present and transported in water bodies are colloids and solid particles.^111,112^

The existence and precipitation mechanisms of rare earth elements in seawater are relatively complex,^113^ and also involve some biological and biochemical processes.^90,114^ Turner and Whitfield^112^ have considered the behavior of the REE in seawater and succinctly argued that it is the solid-state chemistry of the REE, but not the aqueous chemistry, that controls their concentrations in seawater.

The C-G-S rock system of Ordos Basin is composed of different types of minerals such as carbonate minerals (calcite and dolomite), sulfate minerals (gypsum or anhydrite), and halide minerals (halite and potassium halite), which were formed in different stages of crystallization and precipitation by evaporation of seawater, but the overall rare earth element distribution curves of the different rocks are basically the same. It indicates that the rare earth elements still enter the sediment or exist in the seawater medium according to a relatively strict ratio; no significant differentiation of REE related to the difference of mineral crystal structure types appeared.

This also indicates to a certain extent that rare earth elements do not enter the crystal lattice structure of minerals in the form of “isomorphism” during the evaporation concentration and mineral crystallization precipitation processes of the C-G-S system formation (at least not its main form of existence). And more likely to be aggregated in the form of particulate matter, present in lattice defects of the main rock-forming minerals, mineral surfaces, or enclosed by subsequent crystallization growth; but the rare earth elements in this depositional system are too “trace”, and the aggregated particles are too small to be observed under a microscope or a scanning electron microscope for their actual existence.

However, in this overall trend of change, the content of REEs in carbonate minerals and sulfate minerals is significantly higher than the content of REEs in pure halite minerals crystallized in the later stage, indicating the crystal structure type of the host mineral has a certain degree of influence on the precipitation of REEs as a whole. The main reason is related to the existence form of REEs in seawater.

Previous studies have shown that when REEs exist in a dissolved state in seawater, they mainly exist in the form of complexes (chelates) with CO_3_^2–^ and SO_4_^2–^ anions.^103^ So when CO_3_^2–^ and SO_4_^2–^ ions combine with Ca^2+^ and Mg^2+^ ions to form carbonate and sulfate minerals, it must have an impact on the stability of the complexes formed by REEs and CO_3_^2–^ and SO_4_^2–^. Then, when carbonate and sulfate minerals are crystallized and precipitated, the chelation structure of some REE will also coagulate and precipitate together with the carbonate and sulfate minerals.

While in the crystallization and precipitation process of the later evaporation stage of the formation of halite minerals by the combination of Cl^–^ and Na^+^, it has little effect on the stability of the chelation structure of REEs in seawater, thus leading to the evidently low content of REEs in the pure halite rock formed in the late crystallization stage.

### 5.4 Variations in the REE Content Caused by Dolomitization

Dolomitization is a difficult problem that has plagued the geological community for a lot, and for a long time, and various different models of dolomitization mechanisms been proposed, such as the evaporation-pump model, the seepage-reflux model, the mixed water model, the structural hydrothermal model, the microbial induction model, and so on.^115–121^ Recently, another scholar proposed that dissolution enables the growth of dolomite crystals under near-surface environmental conditions.^122^ New insights into the promotion of dolomitization under variable salinity conditions have also been proposed recently.^123–125^

The early mechanism research focused on the analysis of geological environment, rock body occurrence, petrology, mineralogy, carbon–oxygen isotopes, Fe–Mn–Sr, other trace elements, and so on.^126,127^ Dolomitization is rarely linked to the distribution characteristics of rare earth elements. However, in the past 20 years, more and more researchers are paying more attention to the study of the correlation between the dolomitization process and the rare earth distribution characteristics^128–136^ and some new insights were obtained, such as the rare earth element distribution pattern of the dolomite is basically the same as that of limestone, both which are relatively enriched in light rare earth elements, but there are more obvious differences in Eu anomalies (δEu), such as the evaporative tidal flat dolomite and normally buried marine water dolomite usually have no obvious Eu anomalies or have weak negative Eu anomalies. But in structure-hydrothermal dolomite, it is mostly characterized by a marked positive Eu anomaly,^137–140^ which may be related directly to the special high-temperature conditions and the reductive nature of the deep hydrothermal fluids rich in H_2_S.

Recent geochemical analyses of rare earth elements in dolomite have shown that a deeper understanding of rare earth elements can help to trace and understand the genesis of dolomitestone.^141–143^ In the study of the lithology and geochemistry of massive dolostone successions which pervasively occur in the Late Jurassic-Early Cretaceous carbonates in Eastern Pontides (northeast Turkey), some scholars proposed that the coarse-crystal dolomite and the cemented dolomite were formed in the hydrothermal fluid affected by the late Jurassic magmatic event,^144^ and the rare earth elements in the dolomite are mostly characterized by a significant positive Eu anomaly.

The geochemical characteristics of the rare earth elements and Y in the carbonate mineral phases of the Paleoproterozoic hydrothermal massive sulfide deposits in Australia have also been investigated by other scholars.^145–148^ The hydrothermal-genesis carbonate minerals show distinct differences in their REE patterns compared to those of the preore diagenetic carbonate minerals (calcite and dolomite). The hydrothermal carbonate minerals generally exhibit a pattern of marked LREE depletion, and some samples show a very high positive anomaly of the Eu element. Additionally, due to hydrothermal alteration, there is a wide range of enrichment of divalent Fe and Mn in the form of carbonate minerals.^149,150^ However, the REE pattern of the original diagenetic carbonate minerals (nodular calcite) that have not been affected by hydrothermal fluid shows the pattern of LREE enrichment, which is basically consistent with the trend of the REE pattern of the carbonate rocks in the study area of this paper. Moreover, the occurrence of abundant divalent state-enriched Fe–Mn carbonate minerals such as ankerite and the widespread presence of sulfide minerals^148^ suggest that such hydrothermal fluids should be markedly anoxic, and therefore possibly more prone to causing a higher positive Eu anomaly.

It is generally believed that dolomite is a type of rock that experiences replacement on the basis of lime sediments. The dolomites of the carbonate-gypsum-salt system in this area mainly have two genetic types. One is the evaporative tidal flat dolomitization in the quasi-contemporaneous period (similar to sabkha), and the mud crystal dolomite in the Ma 5^6^ Submember belongs to the genetic type; the other is the seepage-reflux dolomitization in the shallow buried environment near the surface, for example, the fine-grained dolomite of the Ma 4 Member in this area is mainly of this genetic type. Mg^2+^ and Ca^2+^ metal cations are brought in and out during the dolomite metasomatic process. Because the arrangement of Mg^2+^ and Ca^2+^ ions in the dolomite crystal structure tends to be more ordered, accompanied by a recrystallization transformation effect, this will inevitably have a certain impact on the rare earth elements, mainly in the form of “impurities” in carbonate minerals, resulting in the overall content of rare earth elements in dolomite being significantly lower than that in limestone.

It can be seen from Figure 7 that due to the different genetic types, the two dolomites with different structural characteristics of the Ma 5^6^ Submember and Ma 4 Member have certain differences in the REE content and the characteristics of the distribution curve. For the mud crystalline dolomite in the Ma 5^6^ Submember, the diagenetic environment is basically close to the sedimentary environment of the original lime sediments due to the dolomitization in the quasi-contemporaneous stage. Therefore, the migration and change of rare earth elements during the dolomitization process are also not great. The powder-fine crystalline dolomite of the Ma 4 Member had undergone a certain burial diagenesis process during its dolomitization, and there are large differences between the diagenetic medium condition and the seawater environment when the original lime sediments are deposited. Coupled with the relatively stable diagenetic medium environment during this period, dolomite undergoes a slower crystallization rate during dolomitization and is accompanied by certain recrystallization transformations, which lead to large-scale migration of rare earth elements during dolomitization. The presence of end-heavy rare earth element odd-to-even anomalies, especially the positive anomaly of the Yb element, may mean that the diagenetic medium environment had been relatively reducing at this time, resulting in the Yb element with variable valence characteristics in the divalent state (Yb^2+^). There are conditions for entering into the lattice of dolomite crystals in the “isomorphism” form, so the content of Yb in the rock is higher than that of the neighboring rare earth elements Tm and Lu.

The study on REE geochemistry of carbonates in the Middle Devonian Presqu’ ile barrier of Western Canada Sedimentary Basin,^151^ and the study on REE distributions within the Lower Cretaceous dolomites and limestones of Central Tunisia,^152^ all show similar patterns of REE changes during the process of dolomitization, and are also similar to that of Ordovician carbonate rocks in the Ordos Basin. First, the general shapes of the REE patterns were preserved during dolomitization. And second, the total REE amounts are somewhat lowered in dolomites compared to parental limestones. In addition, with the different grain structures of dolomite, its rare earth distribution patterns exhibit certain differences: Fine crystalline dolomites retained the REE patterns of their limestone precursors; In the medium and coarse crystalline dolomites, the precursor REE patterns were apparently altered by large volumes of fluids involved during dolomitization.^151^ This indicates that during the process of dolomitization, with the addition of external fluids and the carrying out of Ca ions and the bringing in of Mg ions, the REEs have indeed undergone further fractionation. However, there is still a lack of in-depth analysis on its mechanism and changing conditions.

### 5.5 Trend of Changes in REEs with the Evolution of Depositional Environment

In the C-G-S system of this area, with the improvement of the degree of seawater limitation caused by the falling of sea level, sedimentary sequence of limestone-dolomite-gypsum-salt rocks appeared in turn, and the total amount of REEs also gradually showed a trend of increasing, among which the chondrite-normalized value of REE in carbonate rocks was mostly between 0.5 and 3, which was obviously enriched in light rare earth elements (LREE); the chondrite-normalized values of anhydrite and brownish halite were mostly above 3, and they also had a more obvious trend of light rare earth enrichment. This shows that with the continuous concentration of seawater, rare earth elements gradually showed a trend of separation from seawater and entering sediments. But this does not mean that the REEs are more likely to enter the crystal lattice anhydrite and halite minerals; it is just that part of the REEs are destroyed with the increase of seawater salinity and their original colloidal complex state; it is just that the destruction of the primary colloidal complex state of some REEs in seawater with the precipitation of carbonate and sulfate in seawater and the increase of seawater salinity. Make the REE in seawater combine with CO_3_^2–^ and SO_4_^2–^, coagulate into impurity particles, and precipitate with sulfate and halite minerals.

It is worth noting that the content of rare earth elements in pure white halite is, on the contrary, the chondrite-normalized value of each element is basically below 1.0, and some heavy rare earth elements (HREE) are close to 0.1, or even lower. The author believes that the reason for this phenomenon should be related to the formation of halite, which may have experienced a “washing salt and removing impurities” process in which the early halite layer “recirculated and redissolved” and then recrystallized rapidly due to the short-term sudden change of sea level.^86^ Because according to the rules of “isomorphism”, REEs with trivalent (and some divalent) as the main component have the most difficulty entering the mineral lattice of halite rock dominated by monovalent cations, so the content of rare earth elements in pure halite is the lowest.

### 5.6 Other Aspects of Possible Influencing Factors

In general, this paper is only a general analysis of the depositional process in the evaporative rock cycle and the dolomite process in the early diagenesis period, and does not discuss the effects of hydrothermal fluids and chlorinated groundwater in the middle and late diagenesis stages. However, it is worth noting that previous studies on hydrothermal fluids,^153–156^ and chlorinated groundwater^157^ in the middle and late diagenesis stage have shown that in local areas. These special geological factors can indeed have a great influence on the geochemical characteristics of rare earth elements under certain specific geological conditions. Therefore, in the analysis of the general geological law of rare earth elements in the process of depositional evolution, it is necessary to consider these special geological factors appropriately to correct their individual effects on the distribution pattern of REEs in the general depositional process.

## 6 Conclusions

1. This study investigates the geochemical behavior of Rare Earth Elements (REEs) in the Ordovician carbonate-gypsum-salt rock system (C-G-S system) of the mideastern Ordos Basin. The findings reveal that despite substantial variances in REE content across different rock types formed during various stages of deposition, the overarching distribution patterns exhibit notable similarities. Specifically, the distribution curves show consistent characteristics, such as enrichment of light rare earth elements (LREEs) and pronounced negative europium (Eu) anomalies, indicative of the geochemical processes operating during sediment formation.
2. Importantly, the transition from limestone to dolomite signifies not only a replacement of magnesium for calcium but also a significant outward migration of REEs. This migration occurs during the dolomitization process, underscoring the dynamic interactions between REEs and the mineral matrix in sedimentary environments. Furthermore, the comprehensive analyses indicate that in the C-G-S rock system, REEs do not predominantly reside within the terrigenous clastic impurities, such as clay minerals, nor are they incorporated into the crystal lattice of the principal rock-forming minerals through isomorphous substitution. Instead, REEs tend to aggregate as fine particles found in lattice defects and on the surfaces of these minerals, later being enclosed by crystals formed during subsequent diagenesis.
3. Overall, this research advances the understanding of REE geochemistry within evaporative marine environments, providing pivotal insights into the sedimentological evolution and its implications for resource exploration in the Ordovician context. Future studies should further examine the spatial and temporal variations of REE distribution in sedimentary contexts, enhancing our understanding of the complex geochemical interactions that define these ancient depositional environments.

## Abbreviations

**C-G-S** system, or **C-G-S** rock system: carbonate rock and gypsum salt rock system
**REE,** or **REEs**: rare earth elements
∑REE: total rare earth elements
LREE: light rare earth elements
HREE: heavy rare earth elements
RFM: rock forming minerals
